# AGING RESEARCH IN CRIMINAL LEGAL SYSTEMS: IMPLICATIONS FOR POLICY AND PRACTICE

**DOI:** 10.1093/geroni/igac059.1679

**Published:** 2022-12-20

**Authors:** Jennifer James, Brie Williams

## Abstract

ADD

